# Pregnancy-associated aortopathy and sudden postpartum death

**DOI:** 10.1007/s12024-023-00606-5

**Published:** 2023-04-05

**Authors:** Roger W Byard

**Affiliations:** 1 Forensic Science SA, Adelaide, Australia; 2 School of Biomedicine, The University of Adelaide, Level 2, Room N237, Helen Mayo North, Frome Road, Adelaide, 5005 SA Australia

**Keywords:** Pregnancy, Aortic aneurysm, Aortopathy, Marfan, Sudden death, Forensic

## Abstract

A 39-year-old woman who had undergone an uncomplicated elective cesarean section for a low-lying placenta collapsed and died the following day. At autopsy, there was dissection of an aneurysmally-dilated thoracic aorta with 400 mls of fluid and clotted blood in the pericardial sac. There were no features of Marfan syndrome or other connective tissue disorders. Histology revealed thinning of the aortic wall with fragmentation of elastic fibers and no inflammation. Vessels elsewhere were normal. This case demonstrates a rare complication of pregnancy that may not present until after delivery with unexpected collapse and sudden death. Predisposing factors include an increased cardiac output, reduction in systemic vascular resistance, an increase in left ventricular muscle mass, and alterations in serum progesterone and estrogen levels which may cause structural changes to the aortic wall. The possibility of syndromic and familial connective tissue disorders should also be considered.

## Case report

Following an unremarkable pregnancy a 39-year-old woman underwent an elective cesarean section at term for a low-lying placenta. There were no complications. The day following hospital discharge she complained of dizzyness and collapsed with loss of consciousness. Resuscitative efforts were to no avail. There was a past history of asthma and gestational diabetes.

At autopsy, the length was 156 cm and the weight was 90 kg (BMI 36.98). There was evidence of recent pregnancy with all surgical sites in order. There were no features of Marfan syndrome or other connective tissue disorders; specifically, the height was 156 cm and there was no arachnodactyly, or sternal or palatal abnormalities. The major findings were limited to the chest with postmortem CT examination showing a hemopericardium with recent resuscitation-related rib fractures involving the 1st and 5th ribs anteriorly on the left and the 2nd and 3rd ribs anteriorly on the right. There were no other significant features with all major organs including the heart being unremarkable.

**Fig. 1:**
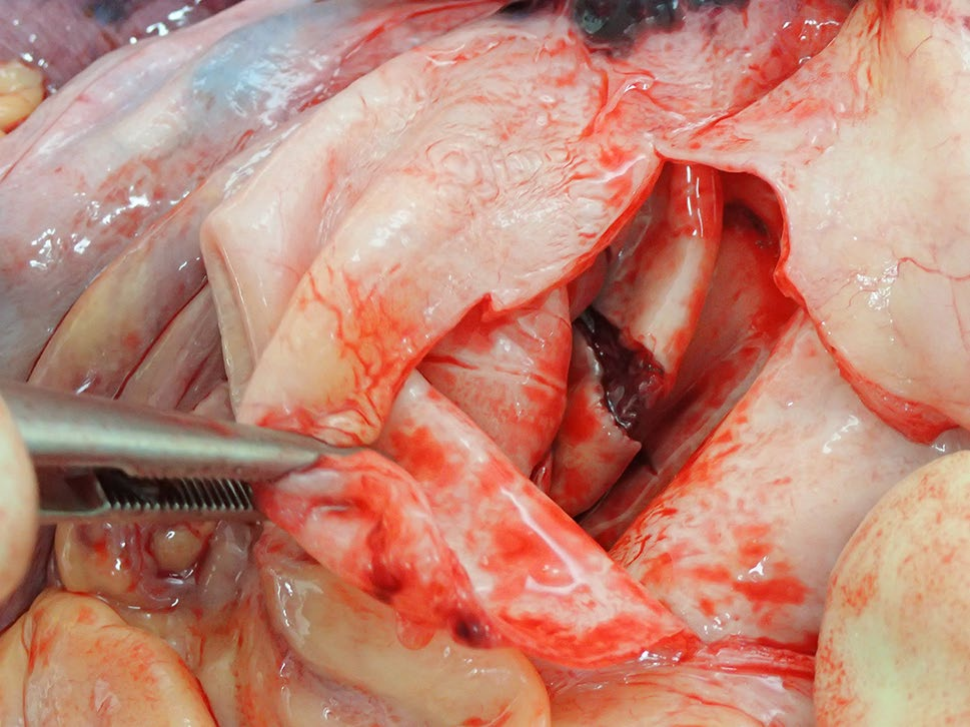
Opening of the aneurysmally-dilated aortic root at autopsy revealed a linear full thickness tear

**Fig. 2:**
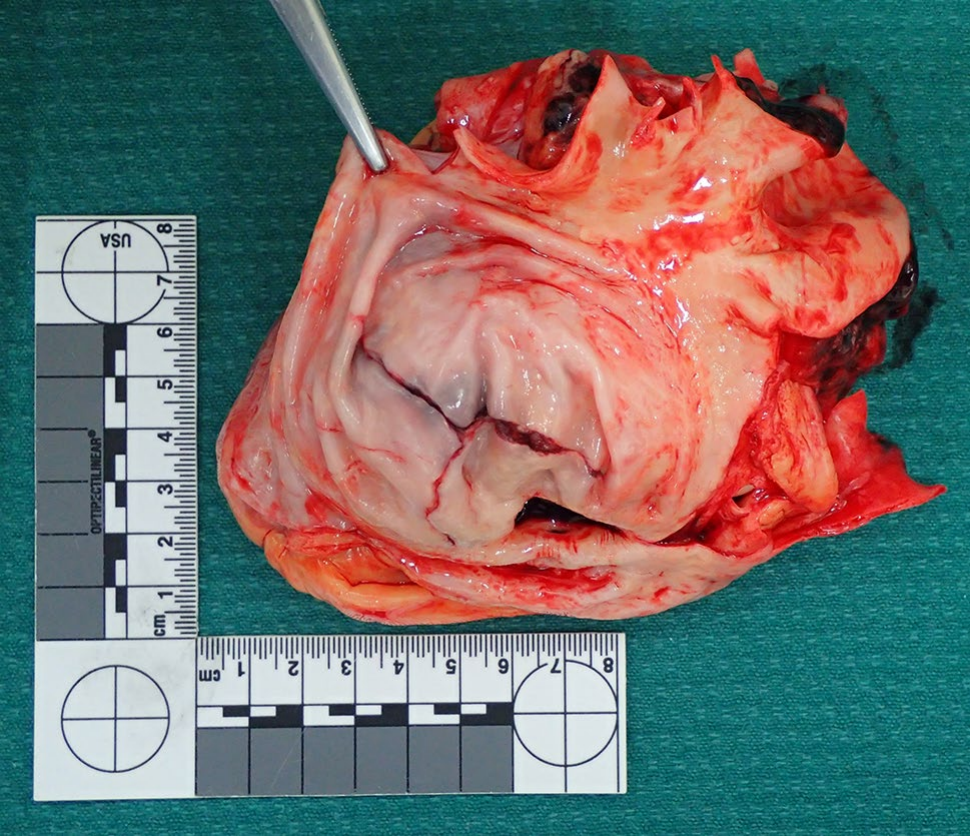
Extending downwards and to the left to a point 20 mm above the aortic valve was a further full-thickness tear measuring 20 mm

**Fig. 3:**
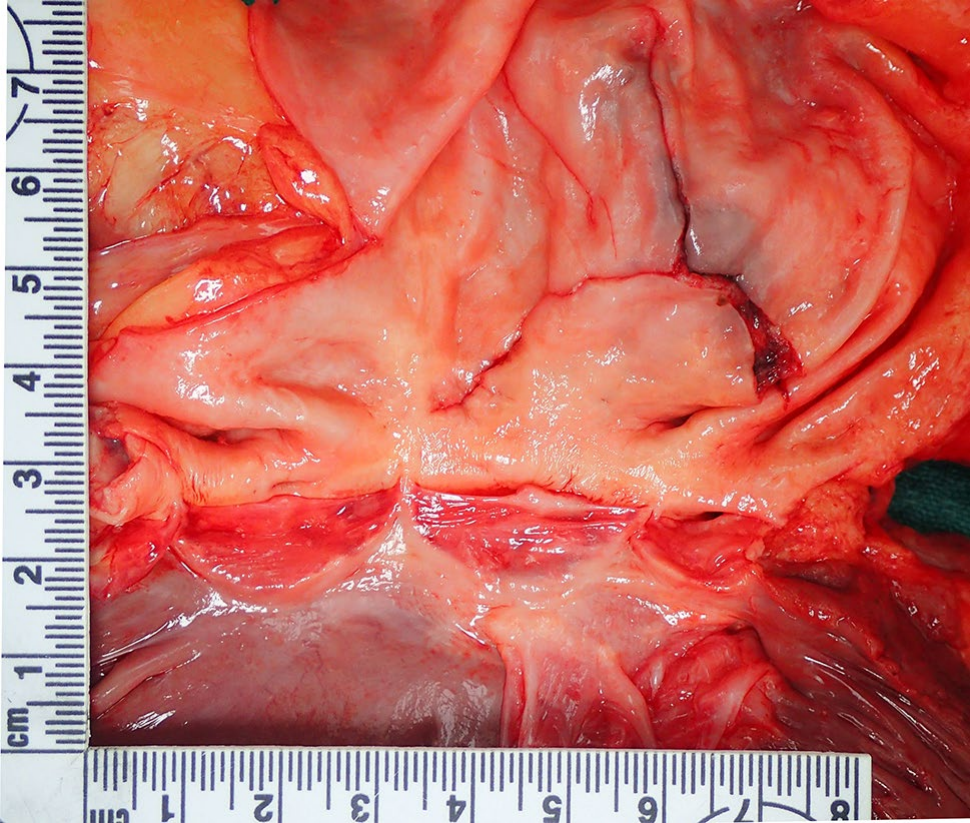
A closer view of the dissection showing three intimal (non-transmural) tears above measuring 30, 15, and 7 mm, respectively

**Fig. 4:**
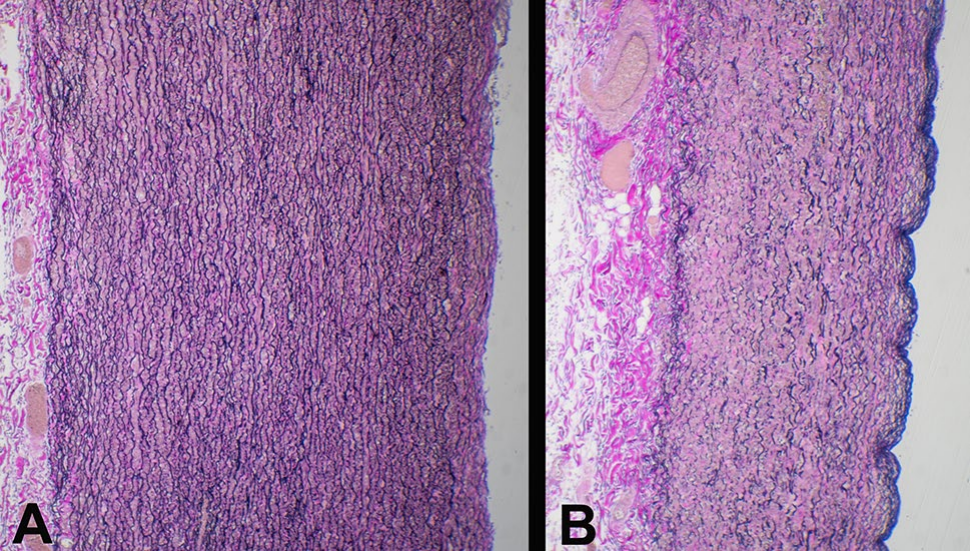
Histology of the aorta demonstrating a section of morphologically-normal distal aorta with regular elastin fibers (**A**) compared to the thinned wall of the aneurysm which showed clear fragmentation of elastin fibers with an increase in background glycosaminoglycans (**B**) (Elastic van Giesen stain × 100)

On opening the chest, a 400-ml hemopericardium was identified composed of fluid and clotted blood. The heart was structurally unremarkable with normal valves. The thoracic aorta (ascending aorta and arch), however, was aneurysmally dilated with a thinned wall. The diameter of the aneurysm was 100 mm and the length was 70 mm. The normal aorta distal to the aneurysm had a diameter of 50 mm. Ten millimeters above the aortic valve, a 65-mm tear was present posteriorly extending distally and to the left (Fig. 1). It was associated with dissection. From the midpoint of this tear, extending downwards and to the left to a point 20 mm above the aortic valve was a 20-mm tear (Fig. 2). Adjacent to the dissection were three intimal (non-transmural) tears measuring 30, 15, and 7 mm, respectively (Fig. 3). Histology revealed thinning of the aortic wall with fragmentation of elastic fibers and no inflammation (Fig. 4). Vessels elsewhere were unremarkable.

The surrounding mediastinum demonstrated interstitial hemorrhage, more on the right than the left sides. There were no other underlying organic diseases present which could have caused or contributed to death. There was no evidence of trauma. Toxicological evaluation revealed no alcohol or significant levels of common drugs. Death was due to a hemopericardium arising from a dissected aortic aneursym in the immediate postpartum period.

## Discussion

Pregnancy has more than 20 times increased risk of aortic dissection with the rate of aortic rupture during pregnancy of 1.39/100,000 woman-years compared to 0.06/100,000 in those who are not pregnant [1, 2]. However, postpartum aortic dissection occurring between day 1 and day 42 after delivery remains a very rare event with only 27 cases reported in the literature from 1988 to 2012 [3].

The age range for aortic dissection in pregnancy is 22 to 39 years with those at highest risk having an aortic root diameter of > 40 mm (as in the present case), a previous dissection and/or Marfan syndrome [3, 4]. Additional risk factors include other connective tissue and genetic disorders such as Loeys-Dietz, Ehlers-Danlos, and Turner syndromes, anatomical anomalies such as bicuspid aortic valve and aortic coarctation, acute myocardial infarction, heavy smoking, obesity, and/or cocaine use [5–8]. A further group of familial aortopathies has been identified which are known as heritable thoracic aortic aneurysm and dissection (h-TAAD). These involve defects in genes that code for extracellular matrix elements, transforming growth factor β (TGFβ) signalling pathways and vascular smooth muscle cell (VSMC) contractile apparatus [9]. In 44.4% of cases, however, no risk factors other than pregnancy will be identified [1, 3]. In the reported case, obesity was the only other risk factor present, with a body mass index (BMI) of 36.98, and no evidence of connective tissue disorders.

Pregnancy predisposes to aortic dissection for a variety of reasons, some of which are hemodynamic associated with an increased stroke volume and heart rate resulting in an increased cardiac output. In addition, there may be reduction in systemic vascular resistance and an increase in left ventricular muscle mass [3]. At the same time, alterations in serum progesterone and estrogen levels may cause structural changes to the aortic wall such as reduction in the amount of acid mucopolysaccharide and elastic fiber disorganization with fragmentation of reticululin [2, 3]. All of this may predispose to structural weakness with aneurysmal dilatation and the potential for dissection. The presentation may be with chest, back, arm, or abdominal pain, or shortness of breath and circulatory collapse [2, 3]. Thoracic aortic dissection has been responsible for 3–14% of maternal cardiac deaths [10]. In the present case, non-specific dizziness was followed by circulatory collapse and sudden death.

This case, therefore, demonstrates a rare complication of pregnancy that may not present until after delivery with unexpected collapse and sudden death. Although a significant percentage of cases may not be associated with identifiable risk factors other than pregnancy, the possibility of syndromic and familial conditions should be considered and family genetic screening and assessment recommended.
